# Illustrations of Infantine Convulsions Arising from Excentric Irritation

**Published:** 1855-04

**Authors:** Robert Molloy


Illustrations of Infantine Convulsions arising from Excentric Irritation. By Robert Molloy, M. D., M. R. C. S.—The convulsive attacks to which young children are so peculiarly liable occasion much anxiety and distress to mothers, and are not unfrequently the source of considerable uneasiness to ourselves. It is desirable, at the outset, to determine exactly what is to be understood by the term convulsion, as it has hitherto been used to include other symptoms which should not fairly be comprehended within it. I would limit it entirely to the involuntary muscular movements, with insensibility, by which it is characterized; and, in order that no doubt may overshadow my meaning, I shall call it by the name of epilepsy, to which it is, without question, most entitled. Moreover, the discrepancy of opinion relative to the treatment to be adopted has been, and is even now, so great, that I am inclined to imagine it must be due, in some measure, to the older nosologists, regarding it as a disease *per se*, instead of simply a symptom, or rather a set of symptoms, arising from some pre-existing cause, sometimes cerebral, but more frequently gastro-intestinal irritation. Attention will be principally directed to the latter in this communication.

The investigation of infantile epilepsy leads at once to the inquiry—what causes are capable of giving rise to this formidable combination of symptoms? And the answer of necessity must be:—the cause most generally assigned by nearly all writers on children's diseases is, dentition. But by no author has this view been more warmly supported, or had, as I consider, such undue prominence given to it, as by Dr. Marshall Hall. My experience leads me to dissent altogether from this opinion; and although in all the cases I have hitherto seen my attention has been particularly directed to this point (not without somewhat of a bias in its favor at the commencement), I have never yet been able to obtain one fact which would corroborate or bear out this view. I am now fully convinced that no permanent relation can be traced between the varying conditions of dentition and the convulsions; neither are the latter removed, or even correspondingly benefited, by any improvement in the state of the former. Other arguments of sufficient weight, and equally convincing, may be briefly adduced. The epilepsy of infants makes its appearance at all stages of dentition; when the teeth are but expanding the jaws, as in the third month; when the gums are soft, cool, with profuse secretion of saliva, and without the slightest indication of irritation; when the gums have been freely lanced, so that no exciting pressure can be present; and even when dentition is wholly completed,—in fact, under all conditions of the mouth and gums.

As, therefore, these varying conditions of dentition bear no constant or real relation to the epileptic convulsions, it is of the highest importance to notice what other morbid symptoms accompany them, in order to trace out the effective cause of so much mischief. This, I believe, will be found almost invariably in the *gastro-intestinal secretions*, which are constantly at fault. My attention was first directed to this subject more particularly by the case of a child, in which I had an opportunity of watching, not only the symptoms present in these convulsive attacks, but all which preceded and followed them. I remarked, on the first epileptic attack to which he was ever subjected, that while under the relaxing influence of the warm bath, an involuntary and very copious offensive discharge from the bowels took place, with *immediate* remission of the convulsive movements. On the second occasion a similar result occurred; and here the change was so instantaneous and striking, from an apparently dying condition to one of promised recovery, that it determined me to adopt the plan of treatment which I have since found singularly successful. This consists in the use of warm water injections, which may be administered to a great extent, and, even in the case of infants, more copiously than any one might believe who has never tried them. I have not unfrequently passed into the bowels twelve syringefuls (about twenty-four ounces) before they could be made to act properly, but in every case with immediate relief, as soon as even a small quantity has been injected. This I have commonly seen:—A child is laboring under fearful convulsive movements; two or three syringefuls of fluid are thrown into the intestines, and on the instant the spasms subside, although but for a short time. I have awaited their return, and again injected more with a like result; but of necessity the patient is not permanently secure from their recurrence until the bowels have been well emptied, all the offensive discharges removed, and a more healthy condition obtained.

The fæcal evacuations in all these cases *contain little or no bile*, and are usually very offensive. In children thus predisposed a daily inspection of the intestinal excreta should take place, as a necessary part of the medical attendant's duty; for I have very frequently been enabled to foretell the approach of these epileptic attacks by the appearances they presented. An additional argument, derived from physiology, may not inaptly be mentioned here. The perspiration of infants is very trifling; their urine, too, is comparatively free from saline constituents. Now, in consequence of the law of compensation, it is probable the greater part of these secretions may have to pass off through the intestines, rendering their contents normally more irritating than, in process of growth, they afterwards become; while the greater relative size of the liver, doing duty, as it were, for the lungs, as well as for itself, compels an incessant functional activity, and requires equally the discharge of bile into, and its speedy withdrawal from, the bowels, to maintain the complete integrity of its functions.

The notes I have taken at different times enable me to cite numberless cases in corroboration of these statements; but I shall content myself with giving outline sketches of a few of them, in which the symptoms were either strongly marked, or had appeared after other plans of treatment had been tried.

A girl, three years of age, semi-idiotic, with a congenitally malformed head, had been subject to frequently recurring convulsive attacks, which, at that time, were referred to the irritation of dentition, and treated in the usual manner by warm baths, incisions of the gums, and application of leeches to the head. One afternoon, at the period when the child was considered unusually well, an epileptic seizure came suddenly on, without any premonitory symptom whatever. Application being made to me, I gave an emetic, which acted in about twenty minutes, ejecting from the stomach a large quantity of undigested salt beef and greens, she had been allowed to eat at dinner. Upon this there was immediate cessation of all convulsive movements; neither did they again return.

Another, four years old, the favored daughter of highly respectable people, who indulged her to an extent which assigned to her omplete mastery over them—a child, fat and gross, with a color n her cheeks that might vie with any country rustic's—was seized n morning, about eight o'clock, with an attack of epilepsy, the first she had ever experienced. The convulsions were certainly the most fearful I have yet seen in one so young; and I was informed that she had been drooping for a few days, was listless and sleepy, but that no aperient medicine had been given for a long time, in consequence of the resistance she made whenever it was attempted. In this instance the effect of the injections was curious and extremely interesting; three or four plunges of the syringe stilled at once, as though by magic, the violent convulsive movements, and although the effect was only temporary, it is of importance to remark, that it was invariably, and always followed by the same calm. It appeared as if the distension of the water removed the surface of the bowels from contact with any irritating matter which was present. As no liquid medicine could be administered by the mouth, I placed some calomel powders on the tongue, and persevered with injections until six in the evening, when the bowels were for the first time relieved, bringing away an enormous quantity of round masses of fæcal matter, *entirely free from bile*, and resembling nothing more nearly than the once favored therapeutic remedy—*album græcum*. These scybala were literally alive with thread-worms, in themselves a most fertile source of intestinal irritation. On this occurrence the convulsions remitted, and shortly afterwards disappeared altogether; nor has she to this date ever had a return.

The third case refers also to a girl, upwards of three years of age, who had completed dentition, and was the only living child of four, the others having died at an early age, either from cerebral excitement or decided hydrocephalus. She was a sharp, precocious child, so that attention would more naturally be directed to the state of the brain in consequence of this circumstance, and of her previous history. The first epileptic attack occurred eighteen months since, at a time when she had been taken a short trip into the country, for change of air, and continued for some hours after she was brought home. She was under treatment longer than usual, principally on account of the time which had elapsed before any continuous plan could be had recourse to. A second epileptic seizure, a few weeks ago, was promptly and decidedly removed by injections. Neither of these attacks differed in any respect from those previously detailed.

The many facts I have witnessed strongly incline me to the belief, that this predisposition to ex-centric epilepsy is hereditary; and one case in particular afforded a very peculiar confirmation of this view. A lady who eventually became my patient, had three children, all girls, none of them of the strongest. They were sorely tried in passing through the customary diseases, but never had any convulsive attack. After being a widow for a few years, she married again, and gave birth to a boy, who suffered long and severely from convulsions. On inquiry, I learnt, not only that the father had been similarly afflicted when he was a child, but that none of the wife's or her first husband's family had ever been subject to them. This was the more conspicuous, because I have generally remarked that girls are more predisposed than boys to epilepsy.

But perhaps the real value of the plan of treatment here advocated was more advantageously displayed in the following case than in those already reported. It made a deep impression upon me at the time, not only on account of the intolerable state of the intestinal secretions in a child of such tender age, but from the immediate and almost instantaneous relief afforded by the injection, which active aperients had failed to obtain. A young child, in whom dentition was complete, was attacked one evening with convulsions, the first he had experienced, and before I could reach the house had two severe fits. A plentiful enema was at once administered, which, in the course of a few minutes, brought away an enormous quantity of extremely offensive evacuations, so that the room was hardly bareable. No return. About two months subsequently, threatening symptoms of convulsion again made their appearance. Purgative medicines were given, by which the bowels were copiously relieved; but, from the persistence of the symptoms, it was deemed advisable to employ an enema, with the immediate effect of emptying the lower intestines of a very great quantity of slate-colored and most unhealthy secretion. He was quite well the next day, and has continued so ever since.

It is of the highest importance, in practice, to dissever from epilepsy, as in the cases already reported, another form of convulsion, which also passes under the term "fits," but it is widely different in its cause, and very frequently, too, in its results: I refer to laryngismus, or as it is more properly described, "spasmodic closure of the glottis." This is in truth a very formidable affection, which appears to run in certain families, and, as far as I have yet been able to observe, is also, in its predisposition, hereditary. In its mildest form, on any little crossing of the child's wishes, any unexpected obstacle or source of irritation, its head is suddenly thrown back, or slight difficulty of breathing, with a gasp, takes place, and recovery occurs almost instantaneously. A more severe kind arises from the same causes, but is mixed up with, and greatly dependent on, obstinacy and a considerable display of temper; in these cases it has always appeared to me that the immediate forerunner of an attack was an unusually prolonged expiration, leading first to spasm of the diaphragm, and then to spasm of the glottic muscles. But it is witnessed in its most severe form, when any concurrent cause has arisen to exalt the sensibility of the muscles of the glottis, going on then to the production of convulsive movements of the eyes, face, and throat, blackness of the face and lips, occasional insensibility, and supervention of complete though temporary asphyxia. I have been much interested of late in watching all these varieties in a family of three young children thus predisposed, two of whom occasioned much anxiety from this unfortunate manifestation of convulsive symptoms, whenever any circumstance transpired to thwart the bent of their inclinations. They were all attacked with hooping-cough, and a more distressing complication could scarcely exist.

A girl, eighteen months old, who had always exhibited the greatest susceptibility as well as the strongest temper, was the first who took the complaint, and the spasms of the throat, with consequent asphyxia, were so violent and frequently recurring, that for many days fears were entertained of the result. Her twin brother was next similarly affected, but it was interesting to observe that it was principally at the commencement of the cough that these attacks were severe. As time waned on, and the children from habit and endurance became better able to manage themselves, retaining their self-possession, and giving way as little as possible to the frequent paroxysms, (a teaching only to be accomplished by time.) they sensibly subsided, and at length entirely disappeared, even though the severity of the hooping-cough had in no respect abated. A constant and anxious observance of these children convinced me how much spasm of the glottis is dependent upon the principle of *imitation.* The boy, a docile and intelligent little fellow, following but too closely the vicious example constantly before his eyes, and being led to believe that such symptoms were a necessary part of the disease he was laboring under, held his breath in like manner, and thus induced the glottic spasms, "because his sister did so." But when extreme watchfulness was exercised over him, and by the judicious combination of firmness with kindness, he was gained over to exert some amount of self-control, by resisting the first approach of all ebullitions of temper which led to these attacks, the spasms ceased altogether in a short time, and occasioned no further trouble. With the girl the case was somewhat different, and involved more difficulty and management. She was of a hasty, impetuous disposition, always a regular Tartar, "*ab ovo ad malum*," and but little amenable to persuasion or correction. Long before the approach of the hooping-cough she had caused much anxiety to her parents by the occasional manifestation of spasms when disappointed of some object on which she had fixed her mind; but when it did make its appearance, and especially at the commencement, they were unusually severe and alarming.

The order of succession in these attacks, as it appears to me, may be succinctly described—mental disappointment; voluntary retention of breath; fixed state of diaphragm, involuntary; with spasm of glottic muscles, and consequent temporary asphyxia. Believing as I do that much of temper is mixed up with these paroxysms, the immediate treatment I adopt consists in dashing a large volume of cold water into the face, so as to give a sudden shock to the system, and to stimulate the inspiratory muscles especially; with the best success, as a general rule, if freely used before the diaphragm becomes absolutely fixed and rigid. And here I cannot forbear to mention a circumstance which tends peculiarly to confirm the view I have taken:—This same child having continued in the best health for two years, on being thwarted by her mother, threw herself into a sudden passion, held her breath as usual, and went off into a severe convulsive fit. It is now about six months since this occurred, and there has been no return to this day.

A most important preventive measure, suggested by the observations already recorded, and which I always endeavor to enforce where possible, is, that children who manifest this unfortunate tendency should associate as little as may be with those of their own or of a younger age. We all know how very soon the young imitate a bad example; the more vicious it is; the more quickly is it learned, and it is not too much to assert that in many cases squinting and stammering, as well as other infantile infirmities, have been acquired, solely owing to the powerful and instinctive imitation of those by whom they are surrounded. While, in adults, hysteria and its allied affections could furnish many corresponding examples, perhaps were the inquiry fairly entered upon, the instinct of imitation might be recognised as a more fertile source of disease than we are at present prepared to admit. At all events its influence is not wholly disregarded by practical men; for, to draw an argument from another and inferior department, I believe no man who keeps a stud of horses would allow a "crib-biter" to enter his stables on any pretext whatever. It is thought, and not without reason, that the evil example of one might eventually become the practice of all.

Returning, however, to the subject in discussion, I can assert, that although the cause of the spasms was not so immediately traceable to intestinal irritation as in cases of epilepsy, they were obviously much kept up and influenced by the state of the secretions. A bileless evacuation was the sure forerunner of a severe attack of glottic spasm, while the appearance of bile in the motions betokened of a surety a sensible abatement. One remark I cannot help making is the difficulty in these cases of getting the liver to act efficiently, whatever remedies may be administered, and the almost certainty of an aggravation of all the symptoms, unless such sholagogue means be perseveringly employed.

If I have spoken slightingly of the power of dentition in producing convulsions, it is because I have never been able to satisfy myself that it was really an active cause in any one instance. I do not pretend to deny, however, that it may be an occasional source of irritation to the system, of a certain importance, and on that account might be strictly kept in view by every prudent practitioner. It is simply an additional cause—one cause the more—which should not be overlooked. A case in point, combining much interest with instruction, was under the care of Dr. W. Bayle. A young man had an attack of epilepsy; this was traced to an abscess of the alveolar process, arising from a carious tooth. On freely opening the abscess, the convulsions subsided. Again, in a few days, they returned, when, on examination, a portion of bone was found in process of exfoliation, and on this being removed, all irritation ceased, and the patient speedily recovered.

It appears to me to be extremely probable, that over severe or long continued irritation of the extremities of all nerves, sympathetic as well as spinal, may be an efficient cause of convulsions; and I do not hesitate to express my decided conviction, that the majority of cases of adult epilepsy are dependent upon *excentric*, rather than centric, irritations. Should this be accepted as a truth, the natural and necessary conclusion is, that epilepsy is more curable than is generally supposed. Were the exciting causes patiently sought for, and a correspo..ding treatment, "not without hope," to be perseveringly adopted, I cannot but believe the happiest results would respond to this conviction, and one opprobrium of our medical science be at once and for ever removed.

For my own part, I never could be brought to appreciate the value of the opinion, that children do, and should inevitably suffer so much from the physiological process of dentition. It is, at the same time, so universal as well as natural, that the idea of necessarily connecting with it pain and disease, which would not be admitted for an instant if associated with the performance of other functions, bespeaks a want of confidence in the harmonies of Nature, and distrust of her ability to carry to a successful termination her own proper and peculiar work. This opinion would seem to have arisen from the known torture adults often experience on the occasion of the eruption of the wisdom teeth; and, by a curious kind of *à posteriori* reasoning, applying the same facts, with all the supposed gratuitous suffering, to infantile dentition. But nothing can be more real than the difference between these cases, which, in fact, present little or no parallelism. The pain endured by grown up persons arises mainly from the circumstance of the jaw, already occupied, being too small to accommodate the uprising tooth, which thus becomes an instrument of offence, causing the intolerable pressure of bone upon bone, or, rather, of a wedge in bone. With children the case is widely different; there is plenty of space; and, in the earlier periods of dentition, the pressure they exercise by biting forcibly everything within their reach, would seem to be intended to relieve uneasiness rather than actual pain, which latter makes its appearance only when the teeth are about to penetrate the gums; and it is at this time precisely they will permit nothing whatever to approach the mouth, very frequently refusing even the breast. (These facts, strangely enough, are reversed by Drs. Maunsell and Evanson.) The majority of children, moreover, cut their teeth so easily that they make no sign by which their immediate attendants are aware of the circumstance; and if the additional fact be borne in mind, that convulsions in infants who are most subject to them, are not present with each tooth penetration, but only when some additional cause has exalted, for the time, their nervous sensibilities, we may well be permitted to question the intimate relation of cause and effect, so frequently taught to be existing between dentition and epilepsy.

Should it, however, be considered that the teeth are occasioning irritation, and that it is desirable to withdraw the child from the influence of an additional cause, even though it be a minor one, there cannot be the slightest objection to the time-honored operation of lancing the gums. The ordinary gum-lancet I regard as an inefficient and barbarous instrument, which might well be dispensed with, to the improvement of the pocket-case. For some years I have made use of the common bleeding-lancet; by this, when the point is carefully guarded, is gained the additional advantage of one simple incision, cleanly and expeditiously made. Its value is more obviously manifest when two or three teeth are supposed to need extrication; here it may be employed without taking the instrument from the mouth; and as less pain is occasioned by this method than the other, and a smaller incision will suffice, it may all be completed in an instant, almost before the child has time to recover from the momentary surprise occasioned by the inspection of the mouth.

In conclusion, I may be permitted to make a few remarks respecting the value of injections in all diseases requiring their use, as well as in their proper method of administration. As they place in the hands of the surgeon the powerful means of compelling the bowels to act in any given time, from five minutes to an hour or more, as he may deem requisite, it is most desirable this power should be exercised by himself alone. Here the rule, of infinite value in every relation of life, is specially applicable, "If you want a thing well done, do it yourself." That this hint is not wholly unnecessary, the following circumstance will show:— I was once called to see a young man, writhing in dreadful pain from obstruction of the bowels, which had lasted some days, and recommended a large enema to be injected. The reply was, five had already been given, and they were useless. On inquiry, information was soon obtained that they were administered by the female attendant, with the old pipe-and-bladder system, which I thought had long since been discontinued! Fifty more, in addition to these, would have produced an equally valueless result. The principle on which an enema acts, is that of distension; if it be requisite to force the intestines to immediate action, it is necessary to throw up a large quantity speedily, until they react against the force employed; while, if time be no great object, a smaller amount, slowly introduced, will dilute the acrid secretions and ensure the action of the bowels within an hour or so of its administration; with the additional advantage of leaving the intestines in a semi relaxed state for some days subsequently, differing in this respect from purgatives, which often do more harm than good by the injurious constipation they commonly occasion. I consider warm water the best injection which can be used; it is always conveniently at hand, and, while its employment more fairly carries out the principle of distension, on which all enemas act, it does this at the same time more efficiently than any medicated Injections I am acquainted with. Leaving out of consideration the complete cleansing of the larger intestine, the beneficial effects of injections, in reference to the cases above described, may be thus summed up:—1. Dilution of acrid secretions. 2. Distension of the bowel, removing the irritated nerves away from the influence of irritating masses. 3. As a fomentation, by the soothing effect which warmth, with moisture, always produces. 4. As a solvent of fœtid gases, which cause much uneasiness, and invariably arise from the prolonged retention of fæcal matter. Most, if not all, these results are directly obtained by the free administration of warm water, and I regard it not only as the best diluent, the best solvent we possess, but the most effectual means of securing the most prompt action of the bowels, whenever such action is desirable.

I cannot forbear the indulgence of the hope that further research will prove the majority of cases of epilepsy in adults, to be dependent upon similar causes, and that it will at length be brought within the list of curable maladies.—*London Lancet.*

